# 11q23 deletion syndrome (Jacobsen syndrome) with severe bleeding: a case report

**DOI:** 10.1186/s13256-017-1535-5

**Published:** 2018-01-08

**Authors:** Yuko Ichimiya, Yuka Wada, Shinji Kunishima, Keiko Tsukamoto, Rika Kosaki, Haruhiko Sago, Akira Ishiguro, Yushi Ito

**Affiliations:** 1 Division of Neonatology, Center of Maternal-Fetal, Neonatal and Reproductive Medicine, National Center for Child Health and Development, Tokyo, Japan; 2 Department of Advanced Diagnosis, Clinical Research Center, National Hospital Organization Nagoya Medical Center, Nagoya, Japan; 3 Division of Medical Genetics, National Center for Child Health and Development, Tokyo, Japan; 4 Center of Maternal-Fetal, Neonatal and Reproductive Medicine, National Center for Child Health and Development, Tokyo, Japan; 5 Division of Hematology, National Center for Child Health and Development, Tokyo, Japan; 6 Department of Pediatrics, Graduate School of Medical Sciences, Kyushu University, 3-1-1 Maidashi, Higashi, Fukuoka, 812-8582 Japan

**Keywords:** Jacobsen syndrome, Paris-Trousseau syndrome, *FLI1*, Thrombocytopenia, Prenatal diagnosis

## Abstract

**Background:**

11q23 deletion syndrome, also known as Jacobsen syndrome, is characterized by growth retardation, psychomotor retardation, facial dysmorphism, multiple congenital abnormalities, and thrombocytopenia. In 11q23 deletion syndrome, it is often difficult to anticipate the severity of bleeding. We report a neonatal case of 11q23 deletion syndrome with bleeding that was more severe than predicted by the platelet count.

**Case presentation:**

We report a case of 11q23 deletion syndrome in an Asian male newborn with severe bleeding just after birth. The diagnosis of 11q23 deletion syndrome was made prenatally by amniocentesis. An array comparative genomic hybridization analysis revealed a deletion of the 13.0 Mb regions ranging from 11q24.1 to the q terminus encoding *FLI1*. Our patient was delivered by cesarean section and exhibited skull deformities, facial asymmetry, low-set ears, inguinal hernia, flat feet, and crowded toes. He had a low platelet count (45,000/μL) and a coagulation abnormality with a prothrombin time–international normalized ratio of 1.92 and an activated partial thromboplastin time of 158.6 seconds. Bleeding at the site of a peripheral vessel puncture was more severe than expected with thrombocytopenia. The peripheral blood featured two different sizes of platelets containing large α-granules. As a result, he required eight platelet transfusions and two fresh frozen plasma transfusions within 13 days of birth. Massive bleeding was avoided, and cerebral magnetic resonance imaging indicated the occurrence of only petechial hemorrhage.

**Conclusions:**

Our patient with 11q deletion including *FLI1* avoided massive bleeding and serious sequelae because of careful management after prenatal diagnosis. We suggest that prenatal diagnosis and vigilant perinatal care including a cesarean section are warranted for patients with 11q23 deletion syndrome.

**Electronic supplementary material:**

The online version of this article (doi:https://doi.org/10.1186/s13256-017-1535-5) contains supplementary material, which is available to authorized users.

## Background

A contiguous gene syndrome, 11q23 deletion syndrome is characterized by growth retardation, psychomotor retardation, facial dysmorphism, multiple congenital abnormalities, abnormal platelet function, and thrombocytopenia. The syndrome is also known as Jacobsen syndrome and the congenital thrombocytopenia/thrombocytopathy observed in most of the affected patients is considered to be the same as that in Paris-Trousseau syndrome [1]. Thrombocytopenia in patients with 11q23 deletion syndrome was determined to be associated with the hemizygous loss of the friend leukemia integration 1 transcription factor (*FLI1*), which is located in the 11q23 chromosome band and appears to be involved in early hematopoietic, megakaryopoietic, and vascular development [2, 3]. In patients with 11q23 deletion syndrome, the platelet count decreases to less than 50,000/μL and bleeding occurs to varying degrees of severity. Platelet abnormality is highly penetrant in 11q23 deletion syndrome, affecting at least 88.5% of patients with the condition [1]. However, it is difficult to predict the severity of bleeding by prenatal diagnosis or platelet count. Therefore, this factor can interfere with qualified perinatal care and may cause life-threatening events leading to an undesirable sequela. We report here a neonatal case of 11q23 deletion syndrome with bleeding that was more severe than predicted by the platelet count. We avoided massive bleeding and serious sequelae by careful management after prenatal diagnosis.

## Case presentation

**Fig. 1:**
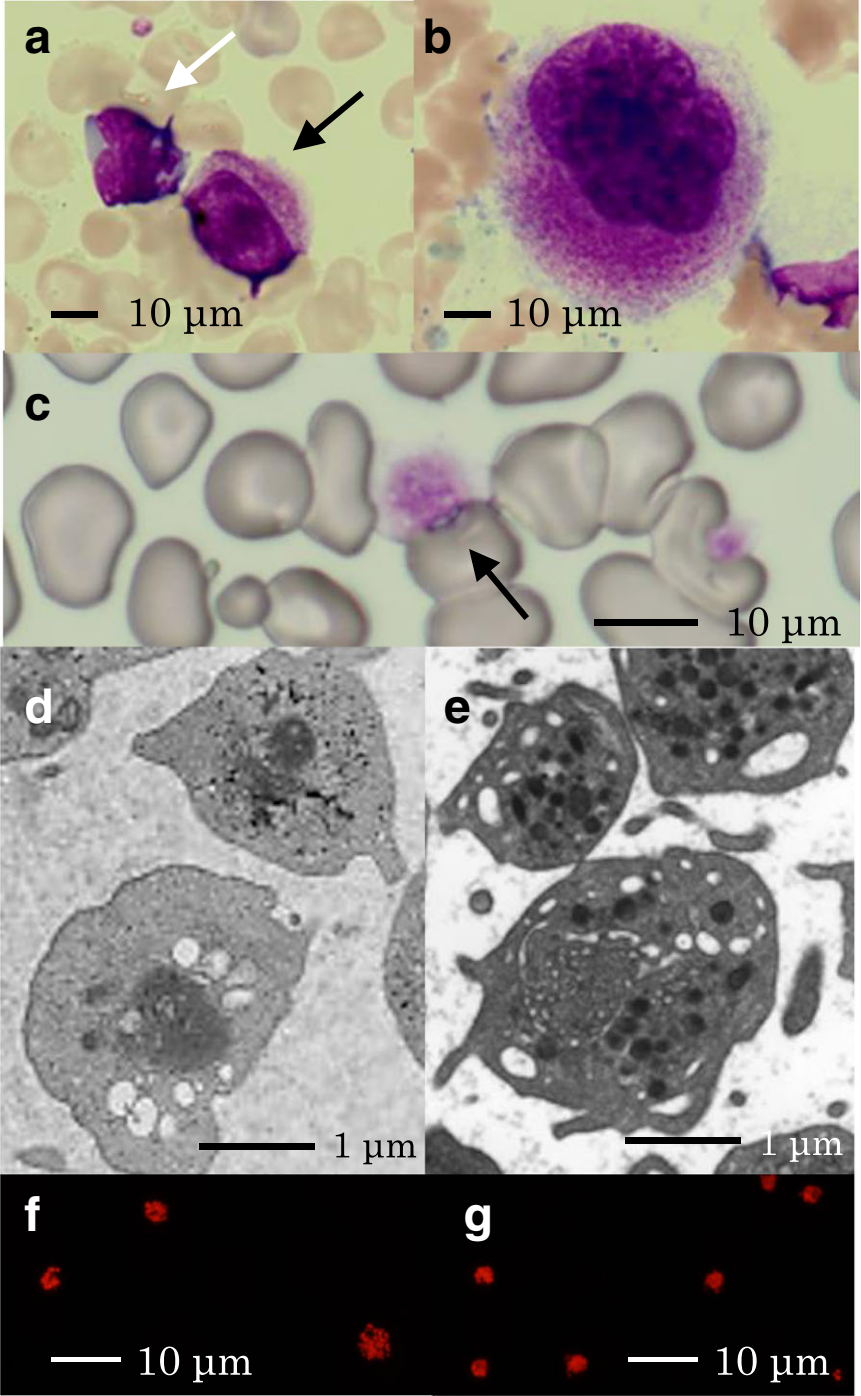
**a**, **b** Megakaryocytes in the patient’s peripheral blood indicated by the *black arrow* (**a**) were smaller than those in the normal control (**b**), which accounted for 150% of lymphocytes indicated by the *white arrow* (May Giemsa stain). **c** Platelets varied in size and included large platelets in the peripheral blood indicated by the *black arrow* (May Giemsa stain). **d**, **e** An electron micrograph showed larger and more heterogeneous α-granules in the patient’s platelets (**d**) than in the normal control (**e**). **f**, **g** α-granules in the patient’s platelets were distributed more sparsely and were more heterogeneous (**f**) than in the normal control (**g**) (thrombospondin-1 stain)

**Fig. 2:**
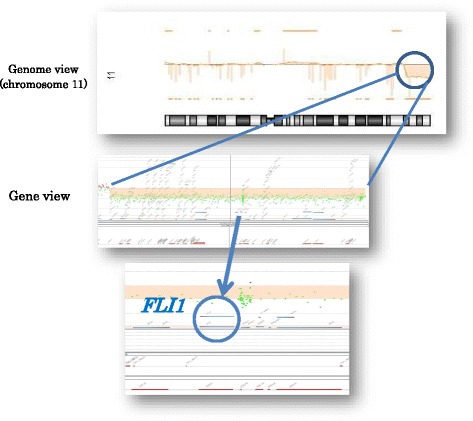
Array comparative genomic hybridization analysis confirmed a deletion of the 13.0 Mb regions ranging from 11q24.1 to the q terminus encoding *FLI1*

A 39-year-old gravida 1 para 1 woman was referred for amniocentesis due to intrauterine growth retardation at 27 gestational weeks. Distal abnormality of chromosome 11: 46,XY, del (q23 > qter) was found. At 36 weeks’ gestation, she went into labor. An Asian male newborn weighing 1082 g with an Apgar score of 5 and 8 at 1 and 5 minutes, respectively, was delivered by cesarean section because of oligohydramnios and a non-reassuring fetal status. In the first hour of life, he developed respiratory failure and was placed on a ventilator. His white blood and red blood cell counts were within the normal range although his platelet count was 45,000/μL with an accompanying coagulation abnormality as indicated by a prothrombin time–international normalized ratio of 1.92 and an activated partial thromboplastin time of 158.6 seconds. He exhibited dolichocephaly with high prominent forehead, facial asymmetry, low-set ears, inguinal hernia, flat feet, and crowded toes. An echocardiogram confirmed hydronephrosis. On capillary blood sampling, bleeding at the site of the peripheral vessel puncture was very severe and required 2 or more hours of oxidized cellulose hemostatic treatment for hemostasis to occur. Since bleeding symptoms were more severe than the thrombocytopenia, he was sedated to avoid intracranial bleeding. Platelets were heterogeneous in size and α-granule morphology was observed in the peripheral blood smears. Some platelets contained giant α-granules that decreased in number (Fig. 1). His platelet count decreased to 18,000/μL on day 6. He required eight platelet transfusions to keep the platelet count above 30,000/μL as well as two transfusions of fresh frozen plasma during the first 13 days after birth to prevent extensive bleeding. He required ventilation until day 11, continuous positive airway pressure until day 54, and oxygenation until day 67. He appeared to have difficulty with oral feeding, but was able to gain the appropriate amount of weight by tube feeding. He was discharged on day 88. At this time, brain magnetic resonance imaging indicated a petechial hemorrhage, which is often seen in neonates, but no hematoma. His platelet count increased to 50,000 to 80,000/μL with normal coagulation although the bleeding time remained prolonged at 6 minutes. An array comparative genomic hybridization analysis confirmed deletion of the 13.0 Mb regions ranging from 11q24.1 to the q terminus encoding *FLI1*, *BSX*, and *BARX2* (Fig. 2).

## Discussion and conclusions

We reported a case of 11q23 deletion syndrome with thrombocytopenia, which showed a greater severity of bleeding than predicted by the platelet count. Our patient had an 11q deletion which includes *FLI1*, a gene associated with dysmegakaryocytopoiesis, *BARX 2*, candidate gene for the development of facial dysmorphism and craniosynostosis, and *BSX*, a gene having a role in regulating locomotor behavior during development.

Thrombocytopenia in 11q23 deletion syndrome can be complicated by platelet dysfunction or coagulopathy, such as in this case, leading to bleeding that is more severe than expected. When 11q deletion occurs, and particularly when it includes *FLI1*, clinicians should be mindful of the risk of bleeding and should consider more careful perinatal management.

Thrombocytopenia is associated with the deletion of *FLI1* because this gene is a member of the E26 transformation-specific family, which has a fundamental role in megakaryopoiesis, embryogenesis, and vascular development [4]. This same abnormality is seen in patients with terminal deletion of 11q. A recent report suggested that a homozygous *FLI* missense mutation can produce a hypofunctional allele and cause the same platelet granule pathology as seen in Paris-Trousseau thrombocytopenia [2]. One study showed that in vivo Fli1-null mice embryos died during early- to mid-gestation because of intracranial hemorrhage and bleeding that resulted from disruption of the vascular plexus. It was speculated this was caused by attenuation of cell-to-cell adhesion between the endothelial cells that comprise the meninges [5]. Furthermore in vitro, megakaryocytes from Fli-1-null embryos showed a reduced number of α-granules, disorganization of membranes, and a reduced size, which is characteristic of poor differentiation [6, 7]. 

Platelet abnormality is highly penetrant in this syndrome, affecting at least 88.5% of cases [1] although severe bleeding such as intraventricular hemorrhage (IVH) is rarely reported. To date, 25 liveborn patients with 11q23 deletion syndrome diagnosed perinatally have been reported (Additional file 1). Of these cases, 17 presented with thrombocytopenia, three showed morphological defects of the platelets, and only one developed coagulopathy [8–11]. The present patient had coagulopathy accompanied by particularly severe bleeding, which may be associated with preterm birth, low birth weight (small for gestational age, SGA, baby) or neonatal asphyxia.

As with our patient, cases of preterm or SGA tend to bleed severely. For example, Trkova *et al*. reported a SGA case of 11q23 deletion syndrome with gastrointestinal echogenicity suggesting intraluminal bleeding [4]. The patient was born with thrombocytopenia (platelet count of 15,000/μL) that also caused IVH [12]. Furthermore, Malia *et al*. reported a preterm and SGA case of severe bleeding during umbilical line placement, in which the platelet count was 44,000/μL and the prothrombin time–international normalized ratio was 1.3 [13].

Thus, the degree of thrombocytopenia is not always indicative of the severity of bleeding. The present case had more severe bleeding than thrombocytopenia (45,000/μL), which was accompanied by platelet dysfunction and coagulopathy. If our patient had been born by normal vaginal delivery, he would have developed more severe bleeding and IVH due to platelet dysfunction and coagulopathy.

An increasing number of cases of 11q23 deletion syndrome are diagnosed prenatally. Nine out of the aforementioned 25 reported cases were diagnosed prenatally and delivered; four of these cases had thrombocytopenia (Additional file 1). Severe cases with fatal bleeding may also eventually be diagnosed prenatally.

Predicting the bleeding tendency in a fetus prenatally is difficult. Therefore, if an 11q23 deletion is detected prenatally, we suggest gentle and less invasive perinatal management including an elective cesarean section and avoidance of vascular punctures. Further case accumulation is necessary to improve the techniques and approaches for managing severe bleeding at birth in newborns prenatally diagnosed with 11q23 syndrome.

## Additional file

Additional file 1: Cases of 11q23 deletion syndrome diagnosed prenatally and delivered. (DOCX 34 kb)

## Abbreviations

*FLI1*: Friend leukemia integration 1 transcription factor
IVH: Intraventricular hemorrhage
SGA: Small for gestational age
