# Supplementary material for: 11q23 deletion syndrome (Jacobsen syndrome) with severe bleeding: a case report
Source: J Med Case Rep. 2018 Jan 8;12:3. doi: 10.1186/s13256-017-1535-5 (PMC5757304; doi:10.1186/s13256-017-1535-5)
Supplement: Additional file 1: — Cases of 11q23 deletion syndrome diagnosed prenatally and delivered. (DOCX 34 kb) [file 13256_2017_1535_MOESM1_ESM.docx]

*Additional file for:*

**11q23 deletion syndrome (Jacobsen syndrome) with severe bleeding: a case report**

Yuko Ichimiya, Yuka Wada, Shinji Kunishima, Keiko Tsukamoto, Rika Kosaki, Haruhiko Sago, Akira Ishiguro and Yushi Ito

This file contains the following materials:

Supplementary table (Table S1) **“Cases of 11q23 deletion syndrome diagnosed prenatally and delivered”**

- Additional references

Table S1

|  | Prenatal diagnosis | Gestational week | Birth weight | Sex | Thrombocytopenia | Platelet findings | Bleeding tendency |
| --- | --- | --- | --- | --- | --- | --- | --- |
| Schinzel^[1]^ | - | 38w | 3,090g | Male | - | - | - |
|  | - | Unknown | 3,050g | Female | + | - | - |
| Bui ^[2]^ | + | Unknown | Unknown | Female | Unknown | - | Unknown |
| Voullaire ^[3]^ | - | Unknown | 2,750g | Male | + | - | - |
| Fryns ^[4]^ | - | Term | 2,550g | Male | - | - | - |
| Wax ^[5]^ | + | 40w | 3,237g | Male | - | - | - |
| Breton-Gorius | - | Unknown | Unknown | Male | 91,000/ μL | Giant granules | - |
| Clang ^[6]^ | - | Unknown | Unknown | Male | + | - | - |
| Michaelis ^[7]^ | - | Term | 2,165g | Female | - | - | - |
|  | - | 40w | 3,100g | Female | + | - | - |
| Krishnamurti ^[8]^ | - | 35w | 1,880g | Male | + | Giant α granules | - |
| Baena | + | Unknown | Unknown | Unknown | Unknown | - | Unknown |
|  | + | Unknown | Unknown | Unknown | Unknown | - | Unknown |
| Zahn ^[9]^ | - | 39w | 3,130g | Male | 94,000/ μL | - | - |
| Van Zutven ^[10]^ | - | 41w | SGA | Female | + | - | Perioperative bleeding |
|  | - | Term | 3,320g | Male | + | - | - |
| Fujita | - | 37w | 2,050g | Female | 40,000/ μL | Giant platelets | - |
| Chen ^[11]^ | + | 37w | 2,530g | Female | 63,000/ μL | - | Petechiae, Coagulopathy |
| Trkova | + | 38w | 2,740g | Female | - | - | - |
|  | + | 39w | 2,410g | Male | 15,000/ μL | - | Intraluminal bleeding, IVH |
| Johnson ^[12]^ | - | Unknown | Unknown | Male | 100,000/ μL | - | Purpura,Petechiae |
|  | - | Unknown | Unknown | Male | + | - | - |
| Malia | - | 36w | 1,775g | Female | 44,000/ μL | - | Bleeding from umbilical line |
| Lo | + | 39w | 3,174g | Female | 18,000/ μL | - | - |
| Present case | + | 36w | 1,036g | Male | 18,000/ μL | Large α-granules | Bleeding from venous line |

**Additional References:** **[1-12]**

[1]Schinzel A, Auf der Maur P, Moser H. Partial deletion of long arm of chromosome 11[del(11)(q23)]: Jacobsen syndrome. Two new cases and review of the clinical findings. J Med Genet 1977, 14:438-444.

[2]Bui TH, Iselius L, Lindsten J. European collaborative study on prenatal diagnosis: mosaicism, pseudomosaicism and single abnormal cells in amniotic fluid cell cultures. Prenat Diagn 1984, 4 Spec No:145-162.

[3]Voullaire LE, Webb GC, Leversha MA. Chromosome deletion at 11q23 in an abnormal child from a family with inherited fragility at 11q23. Hum Genet 1987, 76:202-204.

[4]Fryns JP, Kleczkowska A, Smeets E, Van den Berghe H. Distal 11q deletion: a specific clinical entity. Helv Paediatr Acta 1987, 42:191-194.

[5]Wax JR, Smith JF, Floyd RC, Eggleston MK. Prenatal ultrasonographic findings associated with Jacobsen syndrome. J Ultrasound Med 1995, 14:256-258.

[6]Clang DR, LaBaere RJ. Jacobsen syndrome: chromosome deletion at 11q23. J Am Osteopath Assoc 1998, 98:551-554.

[7]Michaelis RC, Velagaleti GV, Jones C, Pivnick EK, Phelan MC, Boyd E, et al. Most Jacobsen syndrome deletion breakpoints occur distal to FRA11B. Am J Med Genet 1998, 76:222-228.

[8]Krishnamurti L, Neglia JP, Nagarajan R, Berry SA, Lohr J, Hirsch B, et al. Paris-Trousseau syndrome platelets in a child with Jacobsen's syndrome. Am J Hematol 2001, 66:295-299.

[9]Zahn S, Ehrbrecht A, Bosse K, Kalscheuer V, Propping P, Schwanitz G, et al. Further delineation of the phenotype maps for partial trisomy 16q24 and Jacobsen syndrome by a subtle familial translocation t(11;16)(q24.2;q24.1). Am J Med Genet A 2005, 139:19-24.

[10]Van Zutven LJ, van Bever Y, Van Nieuwland CC, Huijbregts GC, Van Opstal D, von Bergh AR, et al. Interstitial 11q deletion derived from a maternal ins(4;11)(p14;q24.2q25): a patient report and review. Am J Med Genet A 2009, 149a:1468-1475.

[11]Chen CP, Lin SP, Hsu CH, Chern SR, Su JW, Chen YJ, et al. Pure distal 11q deletion without additional genomic imbalances in a female infant with Jacobsen syndrome and a de novo unbalanced reciprocal translocation. Genet Couns 2012, 23:223-229.

[12]Johnson JP, Haag M, Beischel L, McCann C, Phillips S, Tunby M, et al. 'Deletion rescue' by mitotic 11q uniparental disomy in a family with recurrence of 11q deletion Jacobsen syndrome. Clin Genet 2014, 85:376-380.
